# Intelligent Therapy Assistant (ITA) for cognitive rehabilitation in patients with acquired brain injury

**DOI:** 10.1186/1472-6947-14-58

**Published:** 2014-07-19

**Authors:** Javier Solana, César Cáceres, Alberto García-Molina, Paloma Chausa, Eloy Opisso, Teresa Roig-Rovira, Ernestina Menasalvas, José M Tormos-Muñoz, Enrique J Gómez

**Affiliations:** 1 Bioengineering and Telemedicine Centre, ETSI Telecomunicación, Universidad Politécnica de Madrid, 28040 Madrid, Spain; 2 Centro de Investigación Biomédica en Red, Biomateriales y Nanomedicina (CIBER-BBN), Zaragoza, Spain; 3 Institut Universtiari de Neurorehabilitació Guttmann adscrit a la UAB, 08916 Barcelona, Spain; 4 Centro de Tecnología Biomedica, Universidad Politecnica de Madrid, 28223 Pozuelo de Alarcon, Madrid, Spain

## Abstract

**Background:**

This paper presents the design, development and first evaluation of an algorithm, named Intelligent Therapy Assistant (ITA), which automatically selects, configures and schedules rehabilitation tasks for patients with cognitive impairments after an episode of Acquired Brain Injury. The ITA is integrated in “Guttmann, Neuro Personal Trainer” (GNPT), a cognitive tele-rehabilitation platform that provides neuropsychological services.

**Methods:**

The ITA selects those tasks that are more suitable for the specific needs of each patient, considering previous experiences, and improving the personalization of the treatment. The system applies data mining techniques to cluster the patients according their cognitive impairment profile. Then, the algorithm rates every rehabilitation task, based on its cognitive structure and the clinical impact of executions done by similar patients. Finally, it configures the most suitable degree of difficulty, depending on the impairment of the patient and his/her evolution during the treatment.

**Results:**

The ITA has been evaluated during 18 months by 582 patients. In order to evaluate the effectiveness of the ITA, a comparison between the traditional manual planning procedure and the one presented in this paper has been done, taking into account: a) the selected tasks assigned to rehabilitation sessions; b) the difficulty level configured for the sessions; c) and the improvement of their cognitive capacities after completing treatment.

**Conclusions:**

The obtained results reveal that the rehabilitation treatment proposed by the ITA is as effective as the one performed manually by therapists, arising as a new powerful support tool for therapists. The obtained results make us conclude that the proposal done by the ITA is very close to the one done by therapists, so it is suitable for real treatments.

## Background

Acquired Brain Injury (ABI) is defined as brain damage that suddenly and unexpectedly appears in people's life, being the main cause of disability in developed countries [1]. The World Health Organization (WHO) [2] predicts that by the year 2020 Traumatic Brain Injury (TBI) and stroke, the two main causes of ABI, will be within the top five etiologies considering not only the economic cost, but also costs related to Disability-Adjusted Life Year (DALY), that can be thought of as the number of years of normal life lost by the disability.

Globally, cerebrovascular disease is the second leading cause of death and the eighth cause of severe disability in the elderly. The WHO estimated that in 2005, stroke accounted for 5.7 million deaths worldwide, and was the predominant cause of disability, afflicting 30.7 million people. Statistical data shows that after a stroke, one third of patients die during the first month, and 40% of people who recover from the acute phase exhibit a high degree of impairment that decreases their independence and quality of life. Only one third of patients recovers their basic functions and can resume a normal life [3].

The incidence of TBI over industrialized countries is in a range of 200 to 300 per 100,000 habitants, with an average age between 16 to 35 and mostly male [4].

Consequences of an ABI vary between cases and can cause motor, cognitive and behavioral deficits to the patient, disrupting their daily life activities at personal, social and professional levels. The most important cognitive deficits after suffering an ABI are those related to attention, decrease of memory and learning capacity, worsening of scheduling and solving problems capacity, reduction of abstract thinking, communication problems, and also a lack of conscience of their own limitations. These cognitive impairments hamper the path to functional independence and a productive lifestyle for the person with ABI [1].

New techniques of early intervention and the development of intensive ABI care have noticeably improved the survival rate. However, despite these advances, brain injuries still have no surgical or pharmacological treatment to re-establish lost functions [5]. In this context, cognitive rehabilitation is defined as a process whereby people with brain injury work together with health service professionals and others to remedy or alleviate cognitive deficits arising from a neurological insult [6]. The provision of cognitive rehabilitation thus becomes an essential part of the services to manage the complex disablement provoked by ABI, allowing recovery of the altered functionalities and preventing the aging-related deterioration. This is achieved by taking advantage of the plastic nature of the nervous system [7], optimizing its capability of functional reorganization and stimulating the creation of new activation patterns.

Despite the existence of empiric knowledge about the benefits of neuropsychological rehabilitation [8], extending it to most potential users becomes difficult due to important limitations. First, the traditional on-site intervention model requires a neuropsychologist supervising the procedure, to administer exercises and cues, based on patient performance. The cost of this process limits the intensity and length of the treatments, compromising sustainability, accessibility and scalability. Besides, the patient is forced to move to the clinical center, making the duration of the treatment conditional to the patient's availability. Finally, in the neuropsychological rehabilitation field there is an absence of clinical practice guidelines to allow a rational extension of these services. Nevertheless, there is sufficient information to support evidence-based protocols and implement empirically-supported treatments for cognitive disability [9].

Neuropsychological rehabilitation and cognitive stimulation aim to minimize or compensate those cognitive deficits for patients who suffer ABI. Traditionally, treatments consist of exercises with different basis (e.g. cards, puzzles, blocks, images or objects), which are specifically selected from detected deficits after a previous neuropsychological assessment. The use of Information and Communication Technologies (ICTs) to develop tele-rehabilitation and tele-assistance systems allows improving the quality and access to clinical services, helping to break geographical barriers. The main objective of tele-assistance is centered on the patient, facilitating communication at different clinical levels. Moreover, one of the main advantages of using ICTs is the possibility to extend the therapeutic processes beyond the hospital (e.g. patient's home). Finally, a reduction of unnecessary costs and a better costs/benefits ratio are achieved, making possible a more efficient use of the available resources [10-12].

“Guttmann, Neuro Personal Trainer®” (GNPT) [13] is a cognitive tele-rehabilitation platform aiming to provide neuropsychological services by optimizing dedicated time with an asynchronous model, increasing personalization and intensity of treatments. The rehabilitation process is also extended beyond clinical centers, breaking geographical barriers. Besides, it automatically monitors treatments based on established therapeutic criteria, reporting real time results and offering the most suitable therapeutic options, based on the patient's characteristics and evolution. Finally, it allows knowledge extraction for the establishment of clinical practice.

The aim of this work is to design, develop and evaluate an automatic therapy planning functionality, called Intelligent Therapy Assistant (ITA), to help therapists to configure the patients’ treatments in the GNPT platform. In this first study, we have focused on the evaluation of the technical viability and the efficiency of the ITA, trying to demonstrate if the clinical outcomes remain, at least, as good as when using the traditional manual planning in GNPT. Besides, a higher variety in the selection of the rehabilitation tasks is expected, what helps to increase the adherence of the treatment. Decision support systems in medicine have been widely used for the last decades [14], like for example in diabetes care [15], in the prevention of cardiovascular disease [16] or, in general, to improve the quality of medical care [17]. However, there is no evidence in the scientific literature on such systems applied to cognitive rehabilitation processes, neither any algorithm to automatically plan rehabilitation sessions to patients based on the information stored in databases. The decision support system presented in this paper classifies and selects the most suitable tasks for each patient, configuring the optimal input parameters to adjust the difficulty level to each patient's specific needs. Data mining techniques are used to classify similar patients, extracting knowledge from the stored results in the system's database.

### Cognitive rehabilitation using GNPT

#### Rehabilitation process

**Figure 1:**
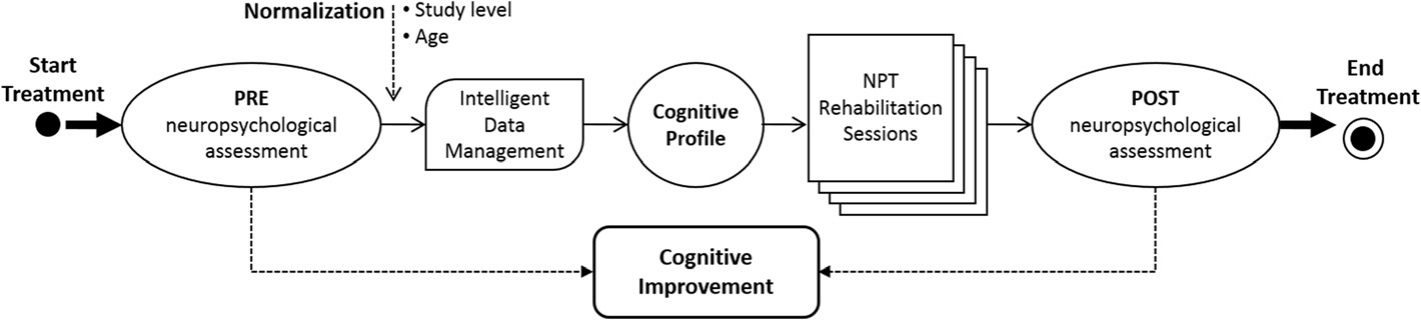
**Rehabilitation process followed using GNPT.** Diagram illustrating the rehabilitation process followed at the Institut Guttmann using Neuro Personal Trainer.

Figure 1 shows the rehabilitation process followed in Institut Guttmann hospital for the cognitive rehabilitation using GNPT.

The process starts by assigning a patient to a therapist responsible for the treatment. Then, the therapist has to perform the initial neuropsychological assessment, consisting of a set of validated tests used to evaluate cognitive functions (attention, memory or executive functions). The results of these tests are stored in the system as the PRE neuropsychological assessment (prior to the treatment), and provide the therapists with information to support their treatment decision. The normalization process and the assignment to a cognitive profile are described in section Clustering of ABI patients.

Usually, treatments consist of 2 or 3 sessions per week, with a total of 60 sessions that last one hour each. The therapist defines these rehabilitation sessions by assigning a set of computerized tasks to a certain day, configuring the input parameters of each task in order to personalize treatments. Once a rehabilitation session is defined, the patient executes the assigned tasks, sending the results back to the server, so therapists can asynchronously see the performance. These results help therapists to select the difficulty level for the next sessions, adjusting treatment to patient evolution.

The system defines three different ranges of performance according to each task’s execution score:

• *Therapeutic* range, when the score is between 65% and 85% of correct answers. The patient executes the task with an appropriate difficulty configuration in order to get the best treatment effectiveness.

• *Infra-therapeutic*, when the score is below 65%. The difficulty level of the task is too high for the patient's capacity and could also lead to frustration.

• *Supra-therapeutic*, when the score is above 85%. The difficulty level is too low for the patient's capacity and the neurological activation is not being high enough. Could also lead to boredom.

These ranges are used by the system to improve the effectiveness of the rehabilitation, by automatically re-launching a task when the score of the patient on that task is out of the therapeutic range, re-adjusting the difficulty level. The objective is to have the patient most of the time executing tasks in therapeutic range, trying to avoid the too easy (supra) or too difficult (infra) ranges during the treatment.

After a patient completes the treatment, the therapist performs the final neuropsychological assessment (POST), which is compared to the PRE one. An improvement of the patient’s cognitive capacities is considered when he or she improves, at least, one of the three main cognitive capacities, and does not get worse in any of the others.

### Cognitive neuro-rehabilitation platform: “Guttmann, Neuro Personal Trainer®”

The “Guttmann, Neuro Personal Trainer®” (GNPT) is a tele-rehabilitation platform developed by a multidisciplinary research team leaded by the Neuropsychosocial rehabilitation area and the research office from the Institut Guttmann, together with the Biomedical Engineering and Telemedicine Centre of the Universidad Politécnica de Madrid. The platform constitutes the second generation of the PREVIRNEC tele-rehabilitation platform [18], which started providing cognitive rehabilitation services in 2008.

**Figure 2:**
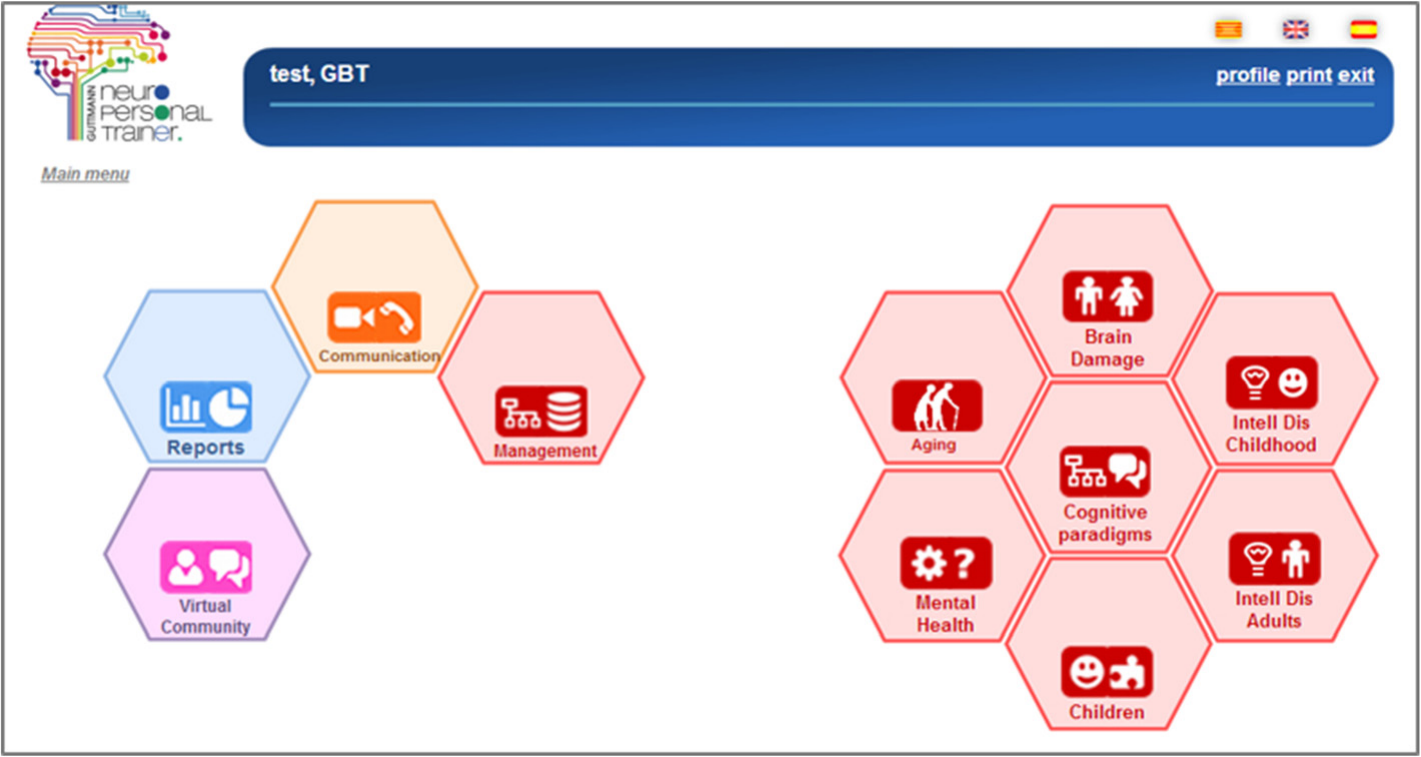
**Main menu of the user interface for therapists, with all the functionalities implemented.** The figure shows an example of the user interface that the therapists see when accessing the system for managing treatments. Each hexagon gives access to a main functionality, like the reports module, or the communication one.

GNPT incorporates multiple technological solutions, from telemedicine services to artificial intelligence applied to knowledge extraction (data mining, collaborative environments, and real-time system adaptation for every single patient). The system is conceived as a tool to enhance cognitive rehabilitation, strengthening the relationship between neuropsychologists and patients, and offering treatment personalization, results monitoring, and computerized rehabilitation tasks performance.This neuro-rehabilitation platform consists of two main different components: on one hand, a web application for therapies management (see Figure 2), where the therapists configure and schedule rehabilitation sessions that consist of a set of computerized tasks; and on the other hand, the client application that patients use to execute the scheduled computerized tasks and send the results to the server. The ITA algorithm has been developed as an innovative functionality for GNPT, helping therapists on their treatment selection and configuration in order to schedule a personalized therapy to each patient.

### Rehabilitation tasks

The rehabilitation content used in GNPT consists of a set of computerized tasks [19], grouped in categories (like ABI), which covers different cognitive functions and subfunctions, as shown in Table 1. Therefore, every task has been specifically designed by neuropsychologists to address a cognitive subfunction, in order to obtain a better personalization of the treatment according to the patient's specific needs. In total, GNPT has 95 tasks designed for ABI.

**Table 1:** Cognitive functions and subfunctions classification for ABI category

**Cognitive function**	**Subfunction**
Attention	Sustained
	Selective
	Divided
Memory	Visual
	Verbal
	Working
Executive functions	Scheduling
	Inhibition
	Flexibility
	Sequencing
	Categorization

**Figure 3:**
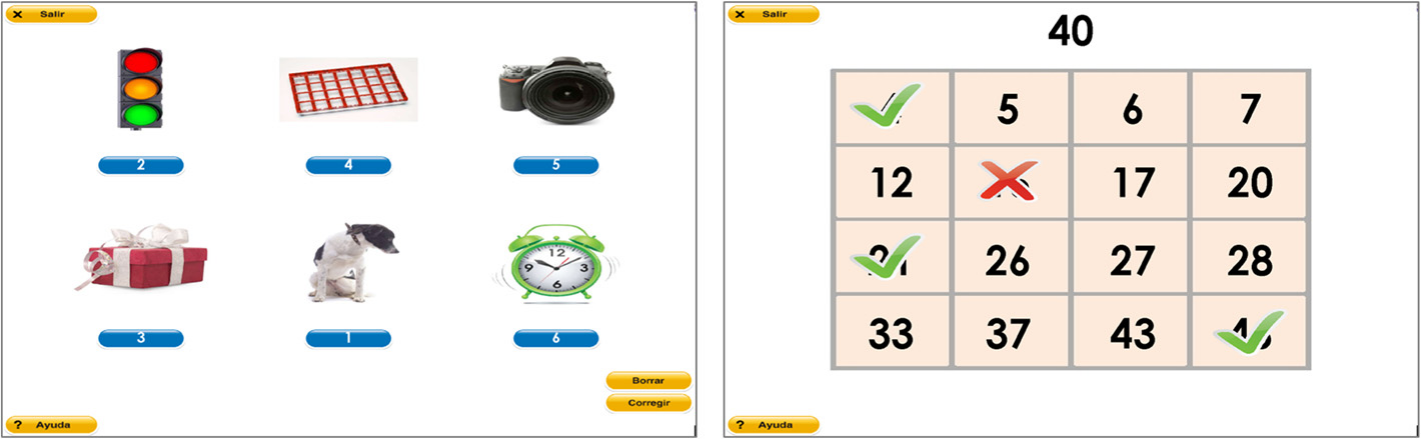
**Neuro-rehabilitation tasks examples.** The figure shows two examples of rehabilitation tasks used in GNPT, for treating working memory (left), and sustained attention (right).

Additionally, neuropsychologists have defined a set of input parameters for every task (e.g. number of images, presentation speed, or latency time), allowing to configure different difficulty levels. Therefore, the treatments can be adjusted to the patient’s specific needs. Besides, they have also defined how the execution result is calculated, based on several performance parameters (e.g. correct and wrong answers, omissions, execution time, etc.) depending on each task. Thus, when a patient performs a task, a score between 0 and 100 is always calculated and assigned to that execution.Examples of two computerized neuro-rehabilitation tasks for ABI patients are shown in Figure 3.In order to help the reader to understand how the ITA algorithm works, the Bingo task is going to be used as an example through the paper. In this task the patient is required to click on the numbers appearing on the screen (see example on the right of Figure 3) and it belongs to the cognitive subfunction “sustained attention”. It has three input parameters, with the following values:

• Dimension of the matrix: “4 × 4”, “5 × 5” or “6 × 6”, representing the number of rows and columns of the bingo card.

• Presentation time: 4, 3.5, 3, 2.5 or 2, meaning the seconds that each number remains on the screen.

• Level: “ordered” or “in disorder”, related to how numbers are spread along the bingo card.

**Figure 4:**
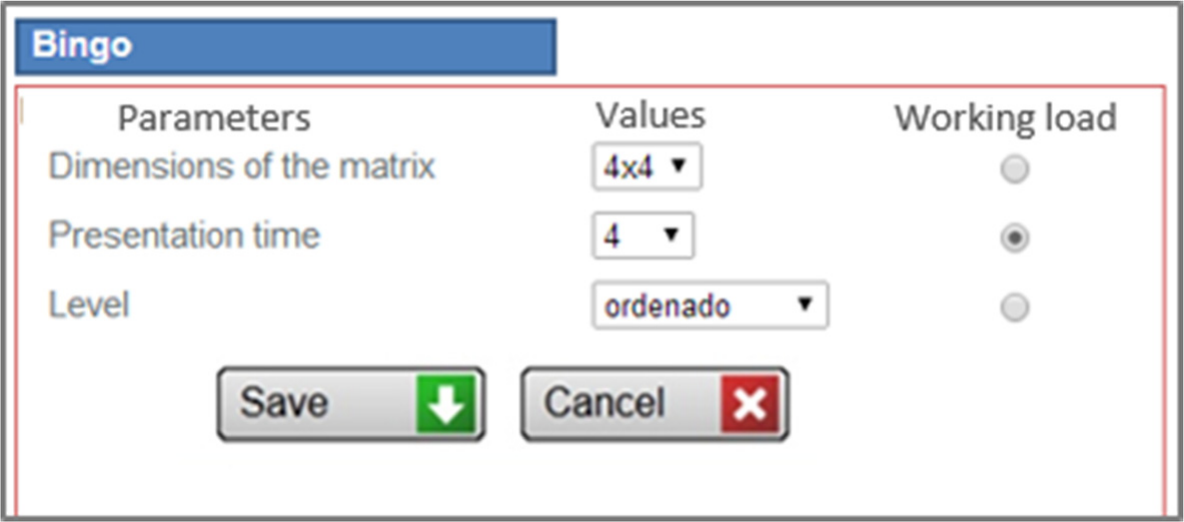
**Input parameters configuration.** Example of interface used to configuring the input parameters of a task, used by therapists to configure the difficulty level when scheduling rehabilitation tasks.

The results defined for this task are the number of correct, incorrect and omitted answers. Thus, the execution score is calculated as the correct answers divided by the total answers, including the numbers omitted.Figure 4 shows the interface used by therapists to manually adjust the values of the different input parameters.

## Methods

### Clustering of ABI patients

GNPT implements a data analysis module able to filter, analyze and extract knowledge from the information stored in the database, in order to aid neuropsychologists in decision-making processes. The use of data mining techniques to predict the outcomes of cognitive rehabilitation in patients with ABI [20] has been revealed as a powerful tool for obtaining new knowledge to evaluate and improve the effectiveness of the cognitive rehabilitation process. Applying data mining techniques to group patients allows us to determine the most suitable therapies for each case, depending on the results and evolution of other similar patients in previous treatments.

In particular, a clustering algorithm has been used to group patients with similar characteristics in order to compare treatments and the evolution of similar patients [21]. The data mining and clustering algorithm has been programmed using the Weka tool (University of Waikato, New Zealand), by implementing the Expectation Maximization (EM) clustering technique. This probabilistic clustering technique is based on a statistical model called Mixture that provides the probability for each patient to belong to a certain cluster.

**Figure 5:**
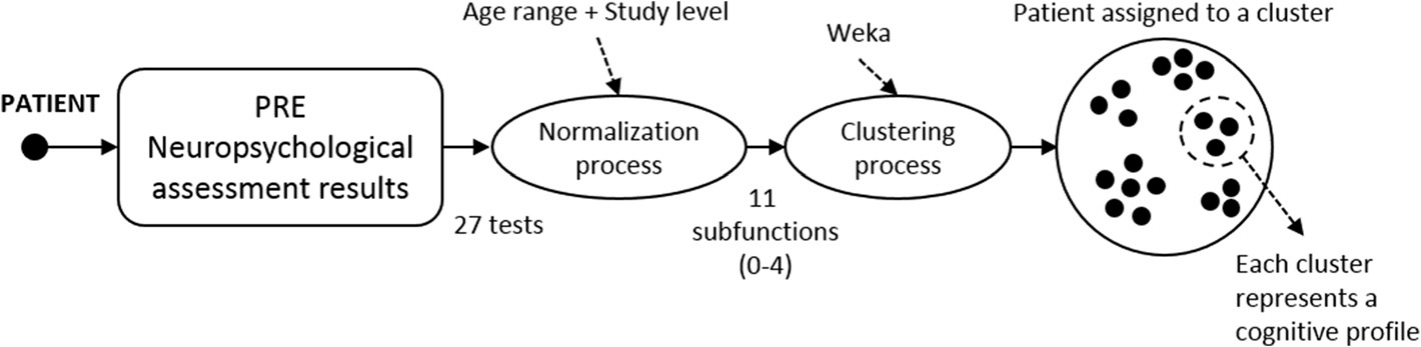
**Clustering process diagram.** Diagram illustrating the different phases of the process followed by the system to assign a patient to a certain cluster, depending on the patient’s neuropsychological assessment and the normalization process that takes into account both the age and study level.

The clustering module assigns a patient to a cluster, depending on his or her cognitive profile. This profile is calculated using the PRE neuropsychological assessment of the cognitive functions, after a normalization process that takes into account the patient's age and study level. Each test’s item has been semantically translated onto the International Classification of Functioning, Disability and Health of the WHO [22], as a common taxonomy to describe patient’s cognitive and functional impairment. As a result, the process rates the 11 defined cognitive subfunctions between 0 (normality) and 4 (very severe impairment) for each patient, resulting on a cognitive profile. The process flow is shown in Figure 5.

Every time a new patient starts treatment using GNPT the clusters are calculated, considering all the information of patients who have already followed a therapy. So, this approach tries to use all the available knowledge in the system related to the PRE tests and the previous therapies and results.

In the end, this clustering process allows the system to group patients with similar characteristics, in order to automatically determine which rehabilitation tasks work better for each cognitive profile, taking into account all previous results and improvements done by similar patients in the past. Moreover, this knowledge can be used to learn about the neuro-rehabilitation processes and to improve the designed tasks, modifying the ones that appear not to be appropriate for certain kind of patients.

### Intelligent Therapy Assistant (ITA)

The Intelligent Therapy Assistant (ITA) algorithm automatically schedules rehabilitation sessions to patients, considering the assigned cognitive profile to determine which tasks are more suitable for their specific needs. The execution results from previous rehabilitation sessions processed by the ITA help the therapist to efficiently personalize treatments according to the patient's characteristics. Naturally, the suggestions provided by the ITA can always be modified by therapists according to their own clinical criterion and experience.

**Figure 6:**
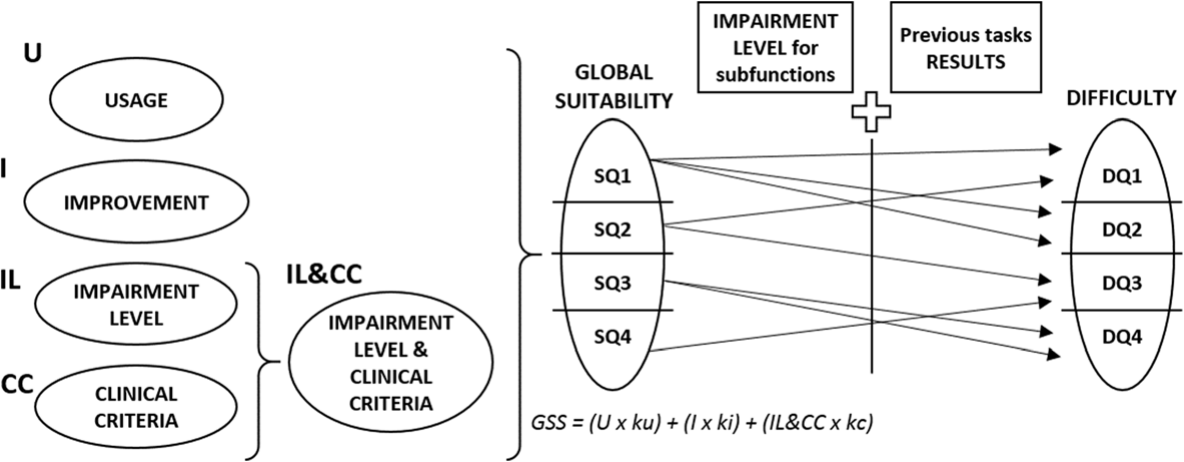
**ITA algorithm diagram.** Diagram illustrating the different scoring criteria and phases used to determine the most suitable tasks to the patient’s specific needs. Then, both the impairment level and the previous tasks results are used to configure the tasks’ difficulty level.

In order to determine the suitability grade for each of the 95 different tasks defined in the system for ABI, the ITA rates every task based on the following scoring criteria:

• *usage score (U),* considering the number of times that the task has been used in other treatments.

• *improvement score (I)*, considering the results obtained by similar patients who executed the task.

• *clinical score (IL&CC)*, as a combination of two different criteria: the *impairment level score (IL)*, considering the patient's initial neuropsychological exploration (PRE) results; and a *clinical criterion (CC)*, considering subjective neuropsychologists’ experience to determine how good a task is to rehabilitate each cognitive function.This scoring process is defined together with a set of variables and coefficients, shown in the equation in Figure 6, allowing the neuropsychologists to adjust the results calculated by the ITA in order to get more realistic configuration results based on their own clinical experience.

Once the scoring process is finished, the system rates all tasks according to their Global Suitability Score (GSS). Then, the system splits these ordered tasks into Suitability Quartiles, from most suitable (SQ1) to less suitable (SQ4). Finally, the automatic therapy planning is done by selecting tasks from the Suitability Quartiles, configuring the appropriate difficulty depending on the rehabilitation needs of each patient.Figure 6 summarizes the process of assigning the score to every task, rating them into suitability quartiles, and how the difficulty level is selected to personalize treatments.

A complete description of the algorithm and its scoring criteria is described next.

#### ***Usage score (U)***

This first criterion gives a score to the task considering the number of executions done by patients with the same cognitive profile. Thus, the used tasks are ordered and divided into quartiles. The algorithm then assigns a score to each task, giving a 4 to the tasks that belongs to the most used quartile, and 1 to the less used quartile, while a 0 is given to the not used tasks.

Consequently, those tasks that have been used more times for similar patients, receive a higher score, rewarding the previously scheduled tasks in GNPT by all the therapists.

#### ***Improvement score (I)***

This second rule rates tasks taking into account the improvement of similar patients who executed the task on the subfunction that particular task was designed for (e.g. sustained attention for the task Bingo). Besides, this rule also considers the improvements that similar patients who executed the task had on the other cognitive functions apart of the one it was designed for (e.g. in the case of the Bingo task, that would be memory and executive functions).

Additionally, thanks to the coefficients defined in the algorithm, neuropsychologists can adjust this rating to promote those tasks that help patients not only to improve the subfunction they were defined for but also the other cognitive functions.

#### ***Impairment level score (IL)***

This score takes into account the patient's previous impairment level (PRE) for every subfunction and function, taking the normalized value of the neuropsychological assessment (from 0 meaning normality to 4 meaning very severe impairment).

The algorithm gives a higher score to those tasks designed for the patient's most impaired functions. On the other hand, if the scoring task is defined for a cognitive function that has less affectation, it receives a lower score.

Using this rule the ITA tries to reward those tasks that belong to the patient's more damaged cognitive functions, because patients need to rehabilitate these impaired functions more than the less impaired ones.

#### ***Clinical criteria score (CC)***

This score determines, from 0 to 4, the suitability of every task to each defined subfunction in ABI. This fourth rule is based on the clinical experience of the neuropsychologists of the Institut Guttmann, who have determined how good is every neuro-rehabilitation task defined in GNPT for the treatment of all the defined 11 subfunctions.

Therefore, a task that has been classified for a certain subfunction can also have a high score for the treatment of other subfunctions, due to their suitability to rehabilitate cognitive capacities in other subfunctions and functions. For example, the Bingo task receives a 4 for sustained attention, 2 for selective attention and 1 for divided attention, while receiving a 0 for all the remaining subfunctions.

#### ***Clinical score (IL&CC)***

This score combines the two previous ones, since they are the most subjective criteria of the algorithm. It also has a coefficient that allows the algorithm to give more or less importance to this combined rule compared to the usage and improvement scores, which are more objective rules.

Table 2 shows an example of the clinical score for the Bingo task, with a particular patient's impairment level and the clinical criteria defined for that task. The Clinical Score is calculated multiplying both subfunction values, obtaining the final score adding them up. So, the Bingo task would receive 19 points according this combined rule.

**Table 2:** Clinical Score example for the Bingo tasks and a patient’s impairment level

**Cognitive function**	**Subfunction**	**Patient’s impairment level (IL)**	**Clinical criteria for bingo (CC)**	**Clinical score**
Attention	Sustained	3	4	12
	Selective	2	2	4
	Divided	3	1	3
Memory	Visual	2	0	0
	Verbal	1	0	0
	Working	3	0	0
Executive functions	Scheduling	1	0	0
	Inhibition	0	0	0
	Flexibility	1	0	0
	Sequencing	2	0	0
	Categorization	0	0	0
			**Final clinical score (IL&CC)**	**19**

#### ***Global suitability score***

Once we have all tasks rated according to the previous three scores, we get the Global Suitability Score (GSS) as a weighted sum of those values. As we can see, thanks to the different coefficients (*kx*) the algorithm's punctuation result can be adjusted to give more or less weight to each of the defined criteria.

$$\mathrm{GSS}=\left(U\times \mathrm{ku}\right)+\left(I\times \mathrm{ki}\right)+\left(\mathrm{IL}\&\mathrm{CC}\times \mathrm{kc}\right)$$

Finally, the system splits all the tasks into suitability quartiles (SQ1, SQ2, SQ3, and SQ4). Then the ITA is ready to automatically create rehabilitation sessions, by randomly assigning tasks from the different four suitability quartiles, until the maximum duration of the session is reached (by default, a rehabilitation session lasts one hour). To do this, the following order is followed: 3 tasks from SQ1, 2 tasks from SQ2, 2 tasks from SQ3 and 1 task from SQ4, and so sequentially. As a result, the algorithm is rewarding tasks from SQ1, but without looking down on the rest of tasks that belong to the other quartiles.

#### ***Difficulty quartiles***

Due to the fact that every computerized task used in GNPT has a set of input parameters to configure the difficulty level, the system assigns a weight to each parameter value, from 0 to n, where 0 means less difficulty. So, each possible parameter values configuration is classified into the Difficulty Quartiles (DQ). The goal is to generate combinations of values to schedule either easy or difficult tasks, adjusting the sessions to the patient’s specific needs. The ITA determines which DQ has to be selected when a task is assigned to a certain rehabilitation session, based on the patient's PRE impairment level.

The ITA schedules sessions in blocks of ten, so for the next ten sessions both the PRE neuropsychological assessment and the results that the patient has already obtained during the treatment are taken into account. This second adjustment criterion parameter is based on the Mean Execution Result for a certain Subfunction (MERS) of the task, which calculates the average result of every already executed task for each subfunction. Thus, the ITA adjusts the difficulty level of the scheduled tasks considering the evolution of the patient, as follows:

• If MERS is in infra therapeutic range (MERS < 65%) the algorithm adds one to the PRE normalized value for that subfunction, considering that the patient needs easier tasks to rehabilitate that function.

• If MERS is in the therapeutic range (65% < MERS < 85%) the ITA subtracts one to the PRE value for that subfunction, considering that the patient is positively evolving and so can do more difficult tasks.

• If MERS is in the supra therapeutic range (MERS > 85%) the ITA subtracts two to the PRE value for that subfunction, considering that the patient can do even more difficult tasks.

This modification considering the MERS comes after an evaluation of the first ITA version, where these patient's execution results were not taken into account. In that previous version, the algorithm scheduled a complete treatment set (normally 60 sessions) instead of blocks of ten. Clinicians saw that the ITA's proposal did not adjust to the patient's evolution during the treatment. As a result, the previous ITA version scheduled tasks at the end of the treatment with a difficulty level lower than the suitable one, so the MERS modification was introduced in the second version.

### Evaluation

GNPT system is running at the Institut Guttmann Hospital in clinical routine, so specific ethical approval is not required to carry out this study. Nevertheless, clinical data usage is aligned with the Declaration of Helsinki, and every treated patient signs the informed consent to participate in the program.

The aim of this evaluation is to evaluate the technical viability and to measure the impact on the efficiency and clinical outcome. So, to evaluate the ITA algorithm, the present study compares the results of the historic manual configuration of sessions performed by therapists to the results once they had the ITA functionality available in the GNPT platform. The ITA has been used for 18 months by 28 different therapists (12 therapists belonging to the Institut Guttmann and 16 therapists from other clinical centers). In total, 582 patients have received treatment using the algorithm presented here, 126 using the first version and 456 using the second one. This means 20,127 rehabilitation sessions automatically scheduled with 92,813 executed tasks. Considering manual planning done by therapists, 1,210 patients have completed treatment, with 44,989 rehabilitation sessions and a total of 286,870 executed tasks.

So, the assessment of the ITA algorithm is focused in the following three outcome parameters:

#### ***Selected tasks for rehabilitation sessions***

In order to compare which tasks are selected for rehabilitation sessions, the number of times that each of the 95 available ABI tasks has been selected has been studied. This will let us know if there are significant differences between the tasks manually selected by therapists compared to those automatically selected by the ITA. A higher variety for the ITA is expected, since the amount of information that a therapist can manage is limited, and they usually schedule the ones that they know the most.

#### ***Difficulty level selected***

The evaluation criteria for assessing the difficulty level configured by the ITA, has been to measure the number of tasks executed in therapeutic range by patients. As it is explained in section “Rehabilitation process”, the system always tries to have patients most of the time executing tasks in therapeutic range, trying to avoid the too easy (supra) or too difficult (infra) ranges during the treatment, and so increasing the effectiveness of the treatment.

At this point, the two versions of the algorithm have been analyzed separately, as we wanted to see the benefits of the improvements introduced in the second one. As it is explained before, the first version of the algorithm scheduled 60 sessions at a time, setting the difficulty level considering just the PRE neuropsychological assessment results. On the other hand, the second version scheduled blocks of ten, taking into account not only the PRE results, but also the patient's evolution to adjust the difficulty of the following rehabilitation sessions.

#### ***Improvement of the cognitive capacities***

A study comparing the improvements achieved by patients after completing treatment has also been carried out. The objective is to see if there are significant differences between the cognitive capacities improvements for those patients that received manual treatment compared to those who received it using the ITA algorithm. Thus, differences between the clinical outcomes will be analysed, letting us to know if the introduction of the ITA into GNPT has undesirable consequences.

So, in order to see the improvements after treatment, a comparison between the PRE and the POST neuropsychological assessment is done, being able to determine the evolution for each cognitive function and subfunction. To carry out the study, we have used a sample of 746 brain injury patients for manual treatment (64% men), while 141 patients have been selected for ITA treatment (55% men). All of them where adults between 16 and 55 years old, with a complete PRE and POST neuropsychological assessment that allows us to see the improvements on the cognitive capacities after completing treatment.

## Results

The results of the first outcome parameter are presented, showing the number of times that each task is selected for a rehabilitation session. Next, how the ITA configures the difficulty level of the rehabilitation tasks is compared, in order to assess which method adjusts better the difficulty according the cognitive affectation level. Finally, a comparison between the improvements of the cognitive capacities after completing treatment is shown, in order to assess the clinical outcomes achieved by the ITA.

### Selected tasks for rehabilitation sessions

**Figure 7:**
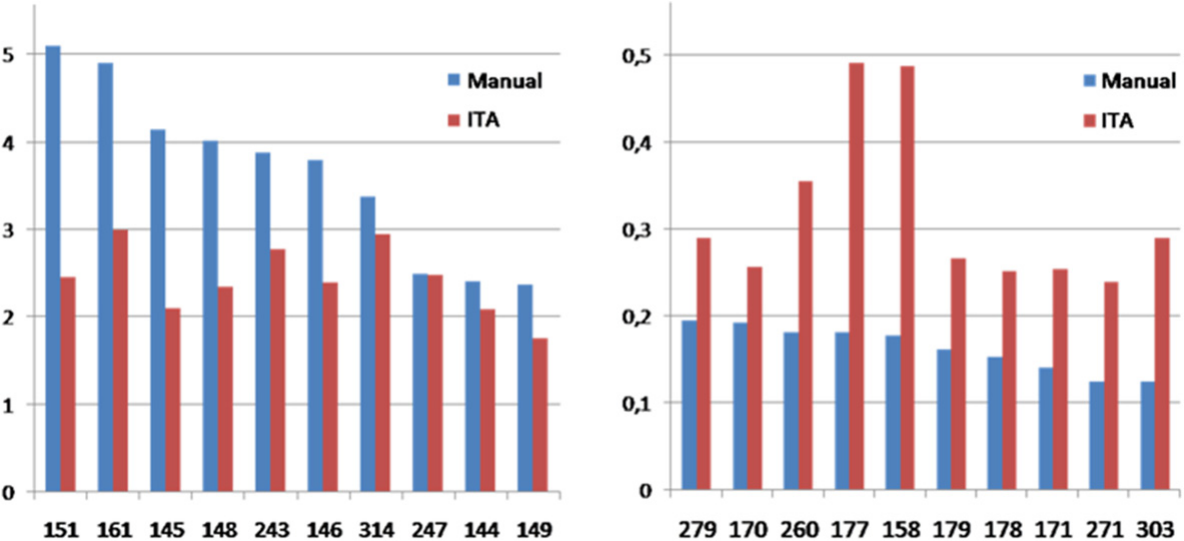
**Tasks selected to treatments comparing traditional manual planning to ITA one.** Blue bars represent the traditional manual planning done by therapists, while red bars show the ITA planning. The left one represents a selection of the most selected ones by therapists, while the right one represents the less used ones by therapists. The y-axis represents the number of times that a task is selected to be assigned to a rehabilitation session, normalized to the total of scheduled tasks, so both data can be compared. On the other hand, the x-axis represents the identification number of the task in the database.

As it is said before, GNPT has 95 different rehabilitation task for treating ABI patients. Figure 7 shows the ITA results considering the number of times that each of these 95 tasks has been selected for a rehabilitation session. In order to compare the tasks manually selected by therapists to those automatically selected by the ITA, results have been normalized to the total number of tasks scheduled, not only the executed one, but also all the selected tasks to be assigned to a rehabilitation session (399,409 for manual planning and 190,197 for ITA planning). So, we can compare the frequency of selection of a task for a rehabilitation session.Figure 7a represents a selection of the most selected ones by therapists, while Figure 7b represents the less used ones by therapists. The y-axis represents the number of times that a task is selected to be assigned to a rehabilitation session, normalized to the total of scheduled tasks, so both data can be compared. On the other hand, the x-axis represents the identification number of the task in the database, so each pair of columns represents a same task.

Besides, there is statistically significant difference (p-value < 0.001) between the manual and the ITA selection of tasks, ensuring that there are differences between the tasks selected by therapists to those ones selected by the ITA.

### Difficulty level selected

**Figure 8:**
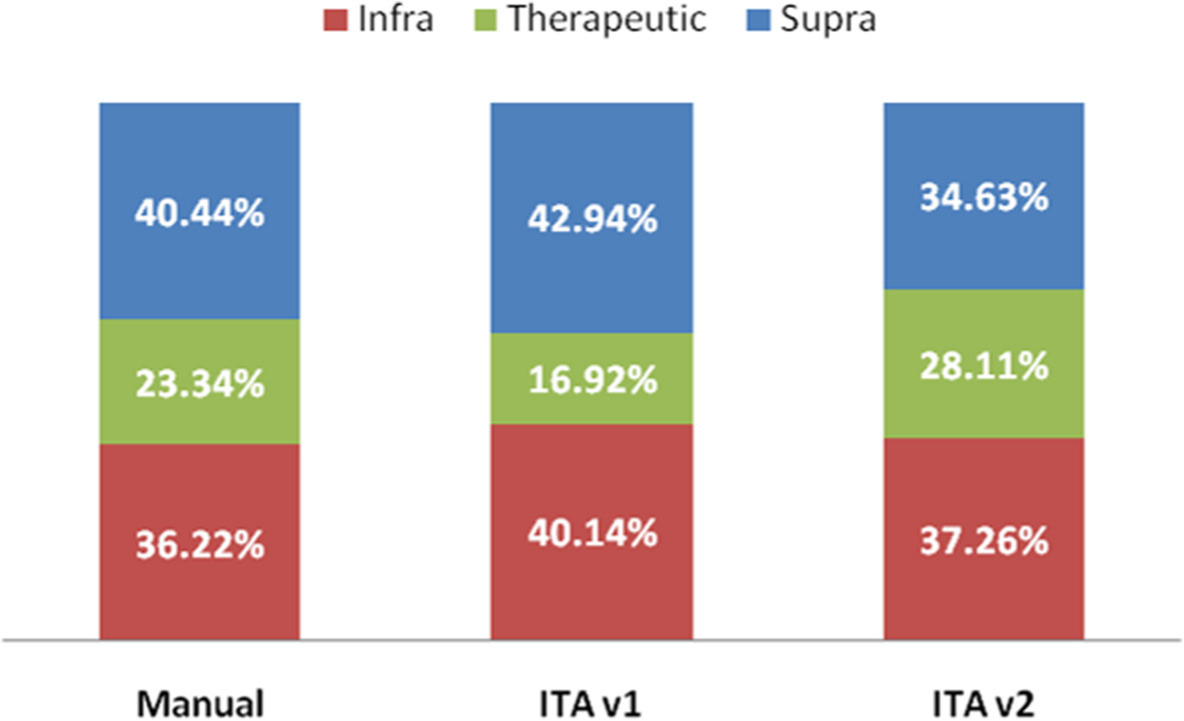
**Execution results tasks ranges comparing manual to ITA planning.** This graph compares the manual planning done by therapists to the automatic one done by the ITA. Take into account that the ITA results are shown distinguishing between the two versions of the algorithm: the first version only considered the patient's PRE assessment results to configure the difficulty level of the scheduled tasks, while the second one also added the patient's evolution during treatment to determine the most suitable difficulty configuration.

In order to assess how appropriate is the difficulty level selected to the assigned tasks, the number of tasks executed in therapeutic range has been studied (the results are shown in Figure 8). This graph compares the manual planning done by therapists to the automatic one done by the ITA. Besides, the ITA results are shown distinguishing between the two versions of the algorithm. Remember that the first version only considered the patient's PRE assessment results to configure the difficulty level of the scheduled tasks, while the second one also added the patient's evolution during treatment to determine the most suitable difficulty configuration.

In order to see if there are significant differences between these results, statistical analysis have been done. After doing the chi-square test for the three samples, it shows a p-value < 0.001, so we can ensure that there are significant differences between the results obtained by the three methods.

### Improvement of the cognitive capacities

**Figure 9:**
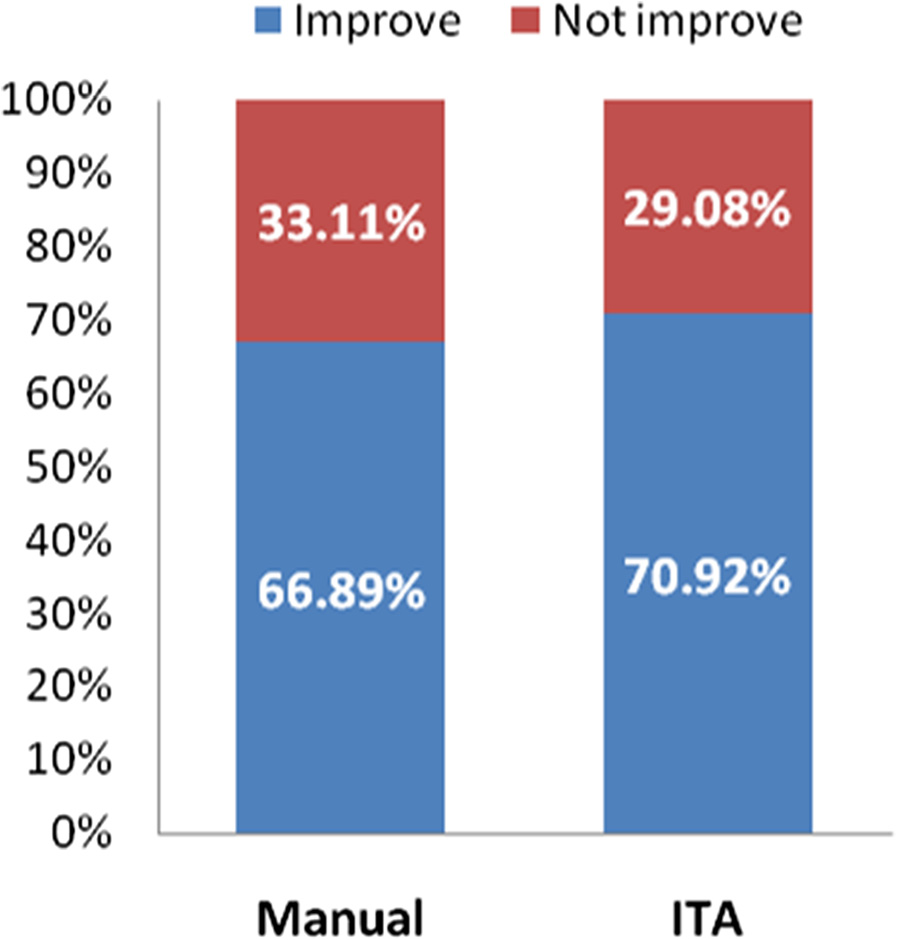
**Patient’s improvement comparison between manual and ITA planning.** This figure shows the percentage of patients who improve their cognitive capacities after completing treatment, comparing the traditional manual planning to the automatic ITA one.

The results of the patients’ improvement after completing treatment are shown in Figure 9. As it is described before, the improvement of the cognitive capacities is calculated comparing the PRE and POST neuropsychological assessment. Once we have this comparison, we consider that a patient improves their cognitive capacities if, at least, he or she improves one main cognitive function and get not worse in any of the others.

Regarding the statistical study, p-value is equal to 0.3484, showing that there is not significant differences between the improvements achieved by each method.

## Discussion

In this study the Intelligent Therapy Assistant (ITA) algorithm has been evaluated, as an integrated functionality in the “Guttmann, Neuro Personal Trainer®” (GNPT) tele-rehabilitation platform. The ITA has been used during 18 months as an automatic tool for the selection and scheduling of therapies for cognitive rehabilitation.Looking at the results for the selected tasks assigned to rehabilitation sessions we see that there are some “favorite” tasks for therapists when planning those sessions; and also the opposite, where some tasks are rarely used to treat patients (Figure 7). Considering that the results of the executed tasks are quite similar, we can say that the ITA is selecting some tasks that are not taken into account by therapists. The same way, the ITA is not giving so much importance to those “favorite” tasks, so we can think that many times therapists select those tasks that they know or like more, and not only those which would work better for the specific needs of the patient. This more equal distribution is achieved thanks to the Improvement and Clinical Scores implemented in the algorithm, compensating the Usage one. So, the ITA also considers the information regarding tasks that could not be properly executed by patients, neither those executions that did not turn into a clinical improvement. This procedure should allow us to refine when a task is selected for a rehabilitation session, beyond the implicit knowledge of the clinicians and their different preferences (knowledge of a task, aesthetic preferences, etc.). Furthermore, the ITA also incorporates the theoretical preferences chosen by consensus of the therapists regarding the suitability of each exercise to rehabilitate each one of the cognitive domains defined in the system (e.g. visual memory or sustained attention). Theoretically, this should lead to a generalization and offer to the patients more varied and better accepted treatments. In this way, the main objective of the ITA and what we try to demonstrate in this work, is the possibility to elaborate a therapeutic plan taking into account all the theoretical premises agreed by clinical consensus. Thus, we offer to the patient more varied exercises and keep, at least, the same level of efficacy than the manual planning, but with lower associated costs and less dependent to the expertise of the therapist (clinical expertise, knowledge of the system, knowledge of the rehabilitation tasks…).

Considering the percentage of tasks executed in therapeutic range comparing therapists (23.34%) to ITA v2 (28.11%) (Figure 9), we can ensure that the difficulty selection procedure performed by the ITA is as good as the one used by therapists. Actually, if we see the results from the two versions of the algorithm, we see that the second one achieves the best therapeutic range percentage. Furthermore, it is desirable to avoid the supra therapeutic range, as we would be trying to treat problems that the patient does not have. Considering this, the second version of the ITA has a 65.37% of executed tasks in both infra and therapeutic range, while the manual procedure has 59.96% and the ITA v1 57.06%. So, we can guarantee that the new characteristics introduced to the second version sensibly improved the results, since the algorithm considers not only the initial PRE assessment to determine the difficulty level of the tasks, but also the patient's evolution during the treatment. However, both therapists and ITA results are quite low, so a deeper study on how GNPT configure the difficulty level has to be carried out to improve the number of tasks executed in therapeutic range. Besides, the different therapeutic ranges are not based on any empiric evidence, but only on general assumptions about which are the generally accepted results desirable to be performed by patients [23]. In this way, we are already planning a deeper study to evaluate this hypothesis.This previous analysis have been done to demonstrate if the ITA algorithm correctly selected the possible parameters values when assigning a task to a rehabilitation session, trying always to have the patient executing tasks in therapeutic range, where the rehabilitation is more efficient. But the final stage of our study is to analyze the differences between the improvements that patients experiment after completing GNPT treatment, comparing those ones treated using the traditional manual planning to those treated using the ITA algorithm. Figure 9 represents the improvement percentage results, where it is shown that there is no significant difference (with a p-value = 0.3484) between the two treatments methods. These results make us conclude that the proposal done by the ITA is very close to the one done by real therapists, so it is suitable for real treatments. However, there is no evidence demonstrating that an improvement in cognitive functions turns into an improvement in Activities of the Daily Living (ADL). In this regard, we plan to introduce ADL questionnaires to assess how the improvement of cognitive functions benefits patient’s quality of life and to introduce this outcome in the proposal done by the ITA.

Besides, the time saved for therapists is quite significant, because they do not need to invest time searching, selecting and configuring tasks, just click a button and wait until the intelligent and automatic process finishes. Then they verify the proposal and modify those tasks and configurations that they do not consider appropriate. After analysing the time expended by therapists in Institute Guttmann using both methods, we have seen that the mean time used for manual planning is about thirty minutes per ten sessions, while by using the ITA the time is reduced to approximately 5 minutes. So, the reduction of time turns into a considerable increase of the efficiency of the scheduling and configuring process. This functionality could also be a good support for a novel therapist, who does not have a high knowledge of every GNPT rehabilitation task, helping them to select the more appropriate tasks for each specific patient.

Looking at the clustering process implemented, we have described how the system dynamically calculates all the clusters when a new patient starts the treatment, instead of assigning a new patient to an already calculated cluster. This way the system ensures that all the clusters are the most suitable to group patients according their cognitive profile, adapting the process to the new patients coming. However, since the variables taken into account to define the clusters are not many, and the amount of patients included in the process is considerably high, we presume that the number of calculated clusters might be tending to stabilization. So, further research must be done in the future, trying to add new clinical variables and also to study the different cognitive profiles defined by the process and their stability. Then, if the clusters are eventually stable, the clustering process might be changed by a classification model.

Besides, another new work is being done, trying to cluster patients based on their results and evolution during treatment. In the coming future, this work will allow us to define new variables to predict how a patient will evolve during the treatment, or even just after the PRE results, by using a prediction model.

## Conclusions

This paper presents the design and first evaluation of an algorithm called Intelligent Therapy Assistant (ITA). This new algorithm automatically plans rehabilitation sessions for patients suffering ABI, who are receiving treatment using the cognitive neuro-rehabilitation platform “Guttmann, Neuro Personal Trainer®” (GNPT). The ITA assigns a score to the computerized neuro-rehabilitation tasks, grouping them into suitability quartiles depending on how good they are for the patient's specific needs.

The ITA is presented as a new powerful support tool for therapists. By managing the high amount of stored data and applying data mining techniques, the ITA extracts information related to the task's suitability to treat each patient depending on his or her cognitive profile. The algorithm has been used for 18 months, with promising results. The improvements achieved by patients in their cognitive capacities after completing treatment using the ITA algorithm are also very similar to the results obtained by using the manual planning. These results make us conclude that the proposal done by the ITA is very close to the one done by real therapists, so it is suitable for real treatments.

## Competing interests

The authors declare that they have no competing interests.

## Authors’ contributions

JS has contributed to the design and implementation of the assistant; to the integration into the tele-rehabilitation platform; to the evaluation of the obtained results; and to the interpretation of the results. CC and PC have contributed to the design of the algorithm; and have supervised the development and evaluation of the assistant. AG has contributed to the design of the assistant; to the clinical validation; and to the analysis and interpretation of the results. EO and TR have contributed to the conception and design of the assistant; and the definition of the evaluation methodology. JMT has contributed to the conception and design of the assistant; the definition of the evaluation methodology; and has coordinated the research work from the clinical point of view. EJG and EM has contributed to the conception and design of the system; has supervised the technical evaluation; and has coordinated the research work from the Biomedical Engineering point of view. JS, CC and PC wrote the first draft of the manuscript. All authors have revised it critically and have approved the final version before submission.

## Pre-publication history

The pre-publication history for this paper can be accessed here:

http://www.biomedcentral.com/1472-6947/14/58/prepub

## References

[B1] Brain Injury Association of America Available online: http://www.biausa.org/ (accessed on January 2014)

[B2] World Health Organization Burden of Disease Statistics Available online: http://www.who.org/ (accessed on January 2014)

[B3] Álvaro LC López-Arbeloa P Cozar R Hospitalizations for acute cerebrovascular accidents and transient ischemic attacks in Spain: Temporal stability and spatial heterogeneity, 1998-2003 J Calid Asist 2009 24 16 23 10.1016/S1134-282X(09)70071-5 19369138

[B4] Tagliaferri F Compagnone C Korsic M Servadei F Kraus J A systematic review of brain injury epidemiology in Europe Acta Neurochir (Wien) 2006 148 issue 3 255 268 discussion 268 16311842 10.1007/s00701-005-0651-y

[B5] Stuss DT Winocur G Robertson IH Cognitive Neuro-rehabilitation: Evidence and Application (Second Edition) 2008 Cambridge, United Kingdom: Cambridge University Press

[B6] Wilson BA Botez MI La réadaption cognitive chez les cérébro-lésés Neuropsychologi e Clinique et Neurologi e du Comportement 1996 2 Montreal: Les Presses de l'Université de Montreal 637 652

[B7] Freitas C Perez J Knobel M Tormos JM Oberman L Eldaief M Bashir S Vernet M Peña-Gómez C Pascual-Leone A Changes in cortical plasticity across the lifespan Front Aging Neurosci 2011 3 Issue APR 1 8 21519394 10.3389/fnagi.2011.00005 PMC3079175

[B8] Carney N Chesnut RM Maynard H Mann NC Patterson P Helfand M Effect of cognitive rehabilitation on outcomes for persons with traumatic brain injury: A systematic review J Head Trauma Rehabil 1999 14 3 277 307 10.1097/00001199-199906000-00008 10381980

[B9] Cicerone KD Langenbahn DM Braden C Malec JF Kalmar K Fraas M Felicetti T Laatsch L Harley JP Bergquist T Azulay J Cantor J Ashman T Evidence-based cognitive rehabilitation: updated review of the literature from 2003 through Arch Phys Med Rehabil 2011 92 4 519 530 10.1016/j.apmr.2010.11.015 21440699

[B10] Lathan CE Kinsella A Rosen MJ Winters J Trepagnier C Aspects of human factors engineering in home telemedicine and telerehabilitation systems Telemed J 1999 5 169 175 10.1089/107830299312131 10908429

[B11] Palsbo SE Bauer D Telerehabilitation: managed care’s new opportunity Manag Care Q 2000 8 56 64 11146846

[B12] Caltagirone C Zannino GD Telecommunications technology in cognitive rehabilitation Funct Neurol 2008 23 195 199 19331782

[B13] García-Molina A Rodríguez-Rajo P Sánchez-Carrión R Gómez-Pulido A Gacría-Rudolph A Solana J Cáceres C Ferre M Roig T Clinical Program of Cognitive Tele-rehabilitation for Traumatic Brain Injury 2010 Warsaw (Poland): eChallenges 1 10

[B14] Welch BM Kawamoto K Clinical decision support for genetically guided personalized medicine: A systematic review J Am Med Inform Assoc 2013 20 2 388 400 10.1136/amiajnl-2012-000892 22922173 PMC3638177

[B15] O’Connor P Sperl-Hillen J Rush W Johnson P Amundson G Asche S Ekstrom H Gilmer T Impact of electronic health record clinical decision support on diabetes care: a randomized trial Ann Fam Med 2011 9 1 12 21 10.1370/afm.1196 21242556 PMC3022040

[B16] Anchala R Pinto MP Shroufi A Chowdhury R Sanderson J Johnson L Blanco P Prabhakaran D Franco OH The role of Decision Support System (DSS) in prevention of cardiovascular disease: a systematic review and meta-analysis PLoS One 2012 7 10 e47064 10.1371/journal.pone.0047064 23071713 PMC3468543

[B17] Kawamoto K Houlihan C Balas A Lobach D Improving clinical practice using clinical decision support systems: a systematic review of trials to identify features critical to success Br Med J 2005 330 775 10.1136/bmj.330.7494.775 15767266 PMC555881

[B18] Solana J Cáceres C Gómez EJ Ferrer-Celma S Ferre-Bergada M García-López P García-Molina A García-Rudolph A Roig T Tormos JM PREVIRNEC A new platform for cognitive tele-rehabilitation Third International Conference on Advanced Cognitive Technologies and Applications (COGNITIVE 2011) 2011

[B19] Caballero R Martínez JM García-Molina A Ferrer-Celma S Solana J Sánchez-Carrión R Fernández-Casado E Pérez-Rodríguez R Gómez-Pulido A Anglès-Tafalla C Cáceres C Ferre-Bergada M Roig T García-López P Tormos JM Gómez EJ 2D-Tasks for Cognitive Rehabilitation. 5th European Conference of the International Federation for Medical and Biological Engineering IFMBE Proceedings 2012 37 838 841

[B20] Marcano-Cedeño A Chausa P García-Molina A Cáceres C Tormos JM Gómez EJ Data mining applied to the cognitive rehabilitation of patients with acquired brain injury Expert Syst Appl 2013 40 4 1054 1060 10.1016/j.eswa.2012.08.034

[B21] Solana J García-Molina A García-Rudolph A Cáceres C Chausa P Roig T Tormos JM Gómez EJ Clustering Techniques for Patients Suffering Acquired Brain Injury in Neuro Personal Trainer ICRAN Conference 2013

[B22] Cieza A Brockow T Ewert T Amman E Linking health-status measurements to the international classification of functioning, disability and health J Rehabil Med 2002 34 5 205 210 10.1080/165019702760279189 12392234

[B23] Morris B Croker S Zimmerman C Gill D Romig C Gaming science: the “Gamification” of scientific thinking Front Psychol 2013 Available online: http://journal.frontiersin.org/Journal/10.3389/fpsyg.2013.00607/full 10.3389/fpsyg.2013.00607 PMC3766824 24058354

